# Conversational health agents: a personalized large language model-powered agent framework

**DOI:** 10.1093/jamiaopen/ooaf067

**Published:** 2025-07-06

**Authors:** Mahyar Abbasian, Iman Azimi, Amir M Rahmani, Ramesh Jain

**Affiliations:** Department of Computer Science, University of California Irvine, Irvine, CA 92697-2625, United States; Department of Computer Science, University of California Irvine, Irvine, CA 92697-2625, United States; Department of Computer Science, University of California Irvine, Irvine, CA 92697-2625, United States; School of Nursing, University of California Irvine, Irvine, CA 92697-3959, United States; Department of Computer Science, University of California Irvine, Irvine, CA 92697-2625, United States

**Keywords:** conversational health agents, large language models, artificial intelligence, open-source agents, openCHA

## Abstract

**Objective:**

Conversational Health Agents (CHAs) are interactive systems providing healthcare services, such as assistance and diagnosis. Current CHAs, especially those utilizing Large Language Models (LLMs), primarily focus on conversation aspects. However, they offer limited agent capabilities, specifically needing more multistep problem-solving, personalized conversations, and multimodal data analysis. We aim to overcome these limitations.

**Materials and methods:**

We propose openCHA, an open-source LLM-powered framework, designed to enable the development of conversational agents. OpenCHA offers a foundational and structured architecture and codebase, enabling researchers and developers to build and customize their CHA based on the specifics of their intended application. The framework leverages knowledge acquisition, problem-solving capabilities, multilingual, and multimodal conversations, and allows interaction with various AI platforms. We have released the framework as open source for the community on GitHub (https://github.com/Institute4FutureHealth/CHA and https://opencha.com).

**Results:**

We demonstrated the openCHA’s capability to develop CHAs across multiple health domains using 2 demos and 5 use cases. In diabetic patient management, developed CHA achieved a 92.1% accuracy rate, surpassing GPT4’s 51.8%. In food recommendations, developed CHA outperformed GPT4. The developed CHA excelled as an evaluator for mental health chatbots, recording the lowest Mean Absolute Error at 0.31, compared to competitors like GPT, Misteral, Gemini, and Claude. Additionally, the empathy enabled CHA identified emotional states with 89% accuracy, and in physiological data analysis of heart rate from Photoplethysmography (PPG) signals, the developed CHA achieved an mean absolute error of 2.83, far lower than GPT-4o’s 8.93.

**Discussion:**

The openCHA framework enhances CHAs by enabling features such as explainability, personalization, and reliability through its integration with LLMs and external data sources. The developed CHAs face challenges like latency, token limits, and scalability. Future efforts will focus on improving planning robustness, enhancing accuracy and evaluation methods, and resolving user query ambiguity to further refine the framework’s effectiveness.

**Conclusion:**

The diverse demos and use cases of openCHA demonstrate the framework’s capacity to empower the development of a wide range of CHAs for various healthcare tasks.

## Introduction

Artificial intelligence (AI), particularly large language model (LLM)-based conversational systems, has attracted immense global attention in recent years. These systems have revolutionized the field by enabling unprecedented access to and interaction with vast amounts of textual information. LLMs can aggregate and process comprehensive or focused segments of textual knowledge existing online, delivering contextually relevant, goal-oriented, and interactive access to this knowledge for anyone who needs it. The advent of LLMs has transformed early, simple conversation systems like Alexa and Siri, demonstrating significant effectiveness across diverse domains.^1–3^ Conversational systems can now engage in open-ended conversations and provide relevant, contextual information in a more natural and engaging way.

While the field of AI has long explored intelligent agents, their focus has primarily been on analyzing the environment and making decisions based on gathered information. Early AI research often concentrated on physical world problems, fueled by advancements in computer vision, audio processing, and other areas of multimodal perceptual understanding. However, in dynamic environments like health management, where personalized and constantly evolving human health states are crucial, intelligent agents need to accurately capture these states through various means, including conversational interactions and access to personal user data. This information needs to be collected and analyzed, leveraging the vast knowledge gathered through research and practitioners’ experience.

Conversational Health Agents (CHAs) hold significant potential to address the challenges of dynamic health management environments. Thanks to the emergence of LLMs, CHAs can now understand user interactions through multimodal conversations, encompassing text, speech, and potentially other modalities. By analyzing these interactions, CHAs need to identify the necessary data, information, computational processes, and knowledge sources required to comprehend the user’s evolving health state. This information is then translated into actionable insights that effectively guide healthcare management. In essence, CHAs should combine the power of LLM-based conversions with agents’ capabilities, leveraging external data and information sources to navigate the complexities of personalized health environments and provide customized support for users:

- *Conversation* is the fundamental mode of human interaction. Throughout the ages, conversations have consistently served as the primary source of knowledge and the catalyst for societal actions. Recently, numerous studies have substantiated the efficacy, usability, and overall satisfaction associated with the conversational aspect of CHAs.^4^ In healthcare settings, *empathy*^5^^,^^6^ and *companionship*^7^^,^^8^ necessitate personalized conversations.
- *Agents* should be furnished with conversational tools, interfaces, computational capabilities, and access to external resources to enhance the quality of healthcare delivery.^9^ Given the intricacies of the healthcare domain,^10^^,^^11^ agents should understand users’ queries, decompose them into the components of knowledge possession, perform health data access and analysis, and apply reasoning to respond effectively to specific situations.^11^ Their adaptability is essential, aligning their evolution with advancements in healthcare technology and literature.^10^ Therefore, agents empower the abilities for *personalization*, *multimodality*, and continuous *up-to-dateness*.^6^

**Figure 1.**
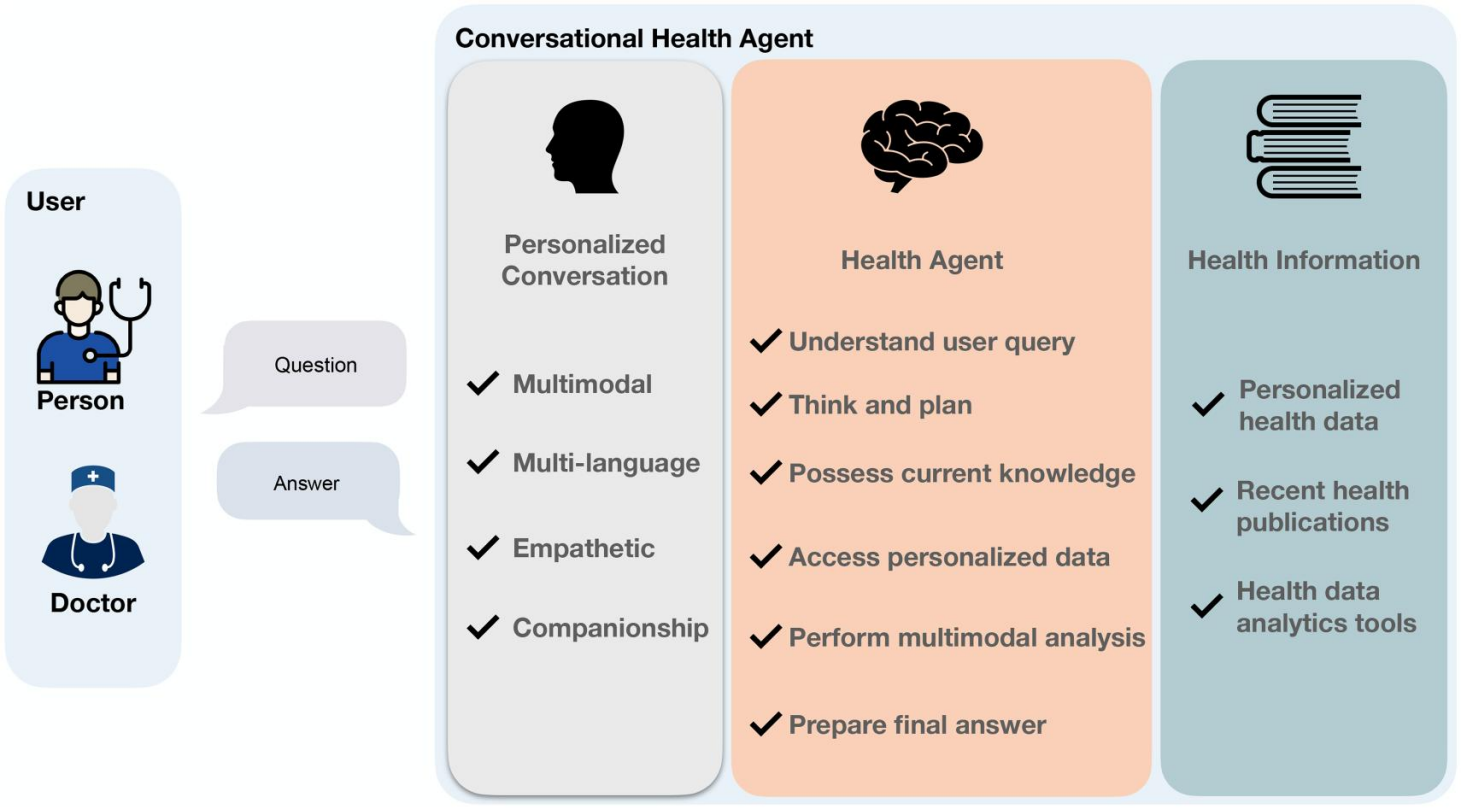
An overview of the CHAs’ main components including (1) a conversation component to enable user interaction and (2) a health agent for problem-solving and determining the optimal sequences of actions, leveraging health information.

Our current exploration centers on the development of CHAs using the latest technological developments in AI, LLMs, and mHealth, where it has shown efficacy in the continuous collection of lifestyle and physiological data from users. Figure 1 shows an overview of the CHA main components. These indispensable components stand poised to facilitate the creation of exceptionally efficient CHAs.

Existing LLMs, such as ChatGPT,^1^ BioGPT,^12^ ChatDoctor,^13^ and Med-PaLM,^3^ are currently active in the medical knowledge domain. These LLMs can be served as CHAs.^9^^,^^14–17^ However, they merely focus on the conversational aspects, offering limited agent capabilities such as basic text-based chat interfaces and lacking multi-step problem-solving capabilities. They lack access to users’ personally collected longitudinal data and electronic health records, which include crucial information like vital signs, biosignals (eg, electrocardiogram), medical images, and demographic data. Consequently, their responses tend to be generic and may not address individual health circumstances adequately. Moreover, they struggle to incorporate the latest health insights, leading to potentially outdated responses.^18^ Furthermore, these chatbots do not seamlessly integrate with established and existing AI models and tools^19^ for multimodal predictive modeling, rendering previous healthcare efforts obsolete.

In light of the significant advancements in technology and its paramount importance for both humanity and the environment, it becomes imperative that we synergize all available tools and harness knowledge from diverse sources to craft CHAs that offer a trustworthy, understandable, and actionable environment for a global audience. Presently, we stand on the threshold of crafting frameworks capable of delivering information in the most user-friendly and culturally attuned manner possible. This paper aims to introduce an initial iteration of such agents and lay the foundation for developing more sophisticated tools as our journey unfolds.

## Background and significance

### Related work

Efforts in developing LLM-based CHAs can be categorized into 3 main groups: LLM Chatbots, Specialized Health LLMs, and Multimodal Health LLMs. LLM Chatbots employ and evaluate current chatbots (eg, ChatGPT) in executing distinct healthcare functions.^20–24^ For instance, Chen et al.^23^ examined ChatGPT’s efficacy in furnishing dependable insights on cancer treatment-related inquiries.

Specialized Health LLMs delved deeper into the fundamental aspects of LLMs, aiming to enhance conversational models’ performance by creating entirely new LLMs pretrained specifically for healthcare or fine-tuning existing models. Notable examples include initiatives such as ChatDoctor,^13^ MedAlpaca,^2^ and BioGPT.^12^ This category emerged in response to research indicating that general-domain LLMs often struggle with healthcare-specific tasks due to domain shift,^25^^,^^26^ and relying solely on prompt engineering may not significantly improve their healthcare-specific performance.^27^^,^^28^

Multimodal Health LLMs involve a novel trajectory by integrating multimodality into LLMs for diagnostic functions. For instance, Tu et al.^3^ investigated the potential of foundational transformer concepts in LLMs to amalgamate diverse modalities—videos, images, signals, and text—culminating in a multimodal generative model. Xu et al.^29^ introduced an LLM-aligned multimodal model, coupling chest X-ray images with radiology reports for X-ray-related tasks. Similarly, Belyaeva et al.^30^ incorporated tabular health data into LLMs, yielding multimodal healthcare capabilities.

### Existing research gaps and challenges

*Knowledge-groundedness* and *personalization* in CHAs require tailored interactions that transcend basic dialogues, ensuring inclusivity through versatile, multimodal multilingual interfaces. The goal is to create CHAs that not only excel in conversational skills but also exhibit agent capabilities, enabling them to engage in critical thinking and strategic planning as proficient problem solvers. Despite the great efforts in developing CHAs, the existing services and models suffer from the following limitations:

1. *Insufficient support for comprehensive personalization, particularly in cases necessitating real-time access to individualized data.* A substantial portion of users’ healthcare data, primarily images, time-series, tabular data, and all other users’ measured personal data streams is housed within healthcare platforms. Currently, CHAs have limited access to this data, primarily during the training and fine-tuning phases of LLM development, or they are completely severed from user data thereafter. The absence of accurate user healthcare information—including continuous data from wearable devices, mHealth applications, and similar sources—hampers the performance of these agents, confining their capabilities to furnish generic responses, offer general guidelines, or potentially provide inaccurate answers.
2. *Limited capacity to access up-to-date knowledge and retrieve the most recent healthcare knowledge base.* Conventional LLMs depend on limited data and Internet-derived knowledge during their training phase, leading to 3 primary challenges. They tend to exhibit biases favoring populations with the most abundant online content, underscoring the importance of accessing the latest, relevant data. Recently introduced LLM-based services (eg, ChatGPT4^1^) offer Internet search, but this is still insufficient for healthcare applications due to the large number of websites propagating false information. They lack updates on newly reliable Internet resources, a critical shortcoming in healthcare where novel, reliable, and evaluated treatments and modifications to previous recommendations are frequently ignored. Lastly, their reliance on outdated or less pertinent data makes identifying instances of hallucination^31^ problematic. Lack of up-to-date information reduces the trustworthiness and credibility of generated responses.^18^
3. *Lack of seamless integration with established, multimodal data analysis tools and predictive models that require external execution.* Current agents often overestimate the computational capabilities of generative AI, leading to an under-utilization of well-established healthcare analysis tools, despite their proficiency in managing diverse data types.^19^
4. *Lack of multi-step problem-solving capabilities.* Existing LLM-based CHAs are typically specialized for specific tasks or deficient in robust data analysis capabilities. For example, Xu et al.^29^ model performs X-ray image reporting relying solely on X-ray images, ignoring other modalities, such as vital signs recorded in time-series format. Additionally, the existing CHAs cannot address intricate sequential tasks (ie, act as problem solvers). Incorporating LLMs into CHAs requires integrating sequential reasoning, personalized health history analysis, and data fusion.

LLMs solely are insufficient to tackle the previously mentioned challenges. To make them practical for real-world applications, we need a comprehensive framework that harnesses LLMs while integrating various auxiliary components and external resources. Table 1 summarizes the existing methods and their limitations.

**Table 1.** Summary of existing LLM-based CHAs.

Category	Description	Limitation
LLM Chatbots	Employ and evaluate current chatbots (eg, ChatGPT) in executing distinct healthcare functions.^20–24^	• Personalization • Up-to-dateness • Multimodal Data Analysis • Multi-step Problem-solving
Specialized Health LLMs	Enhance conversational models’ performance by creating entirely new LLMs pretrained specifically for healthcare or fine-tuning existing models.^13–12^	• Personalization • Up-to-dateness • Multimodal Data Analysis • Multi-step Problem-solving
Multimodal Health LLMs	Integrate multimodality into LLMs for diagnostic functions.^3–30^	• Personalization • Up-to-dateness • Multi-step Problem-solving

### Key contributions

In this article, we propose a holistic LLM-powered framework (ie, OpenCHA) designed to enable the creation of CHAs, aiming to tackle the limitations mentioned above. OpenCHA is *not a specific LLM-based agent or LLM chatbot*. It offers a foundational and structured architecture and codebase, enabling researchers and developers to build and customize their CHA configurations based on the specifics of their intended application. We delineate the components of our framework and perform its capabilities through multiple case studies. The CHAs created in the case studies provide personalized responses by utilizing contemporary Internet resources and advanced multimodal healthcare analysis tools, including machine learning methods. The paper’s significant contributions are as follows:

1. We propose an LLM-powered Orchestrator, acting as a problem solver, to address healthcare-related queries by analyzing input queries, gathering the required information, performing actions, and offering personalized responses.
2. We introduce external healthcare data sources, knowledge bases, and AI and Analysis models critical for enabling CHAs to offer reliable, trustworthy, and up-to-date responses.
3. We incorporate multimodal and multilingual capabilities into the framework, increasing usability.
4. We show the framework’s effectiveness using 2 demos and 4 use cases.
5. We release the CHA framework as open source on GitHub (https://github.com/Institute4FutureHealth/CHA) along with detailed documentation (https://docs.opencha.com/), inviting the community to leverage and integrate it into their solutions.

## Methods

**Figure 2.**
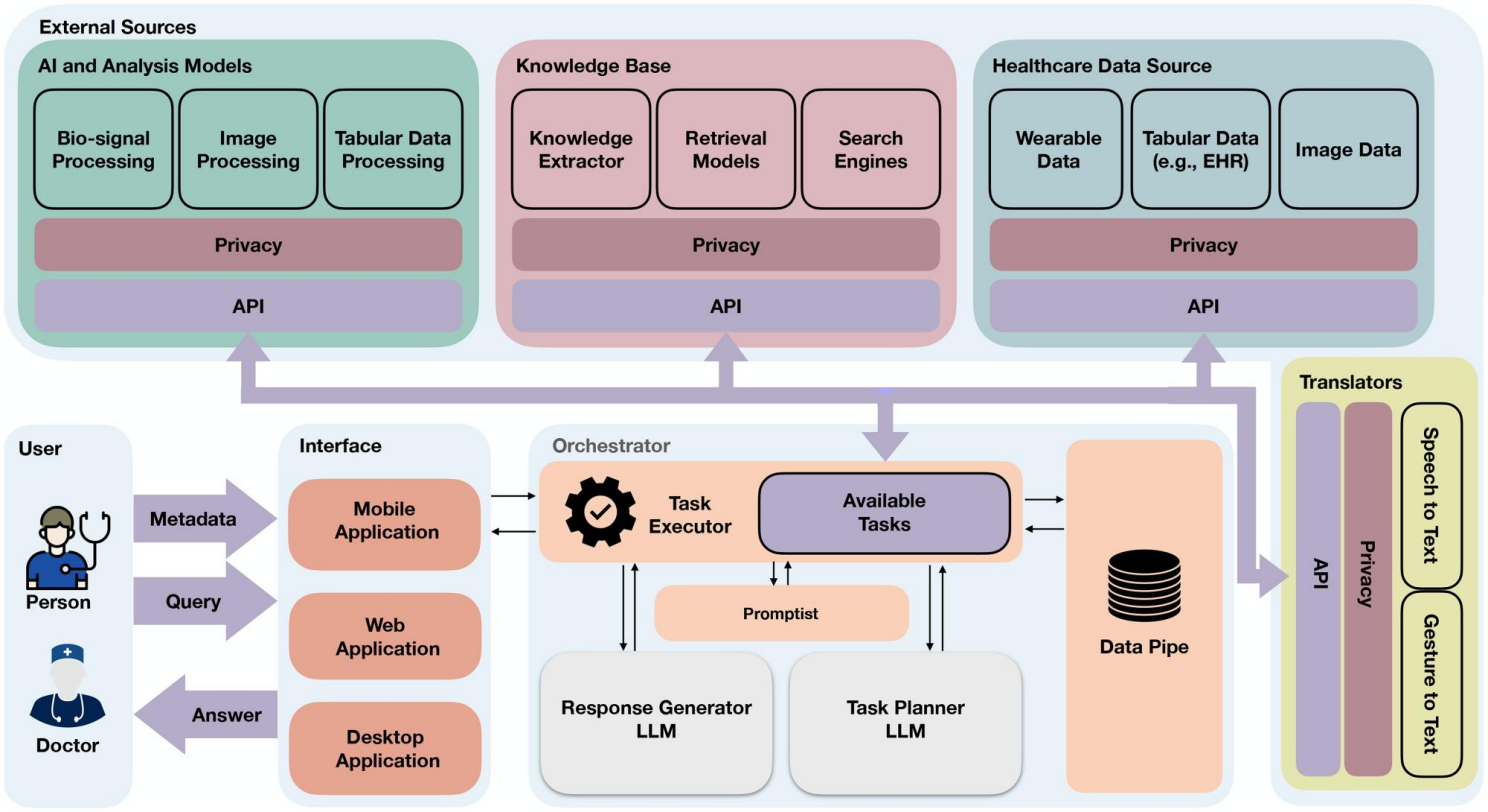
An overview of the proposed LLM-powered framework leveraging a service-based architecture. It includes three core components: Interface, Orchestrator, and External Sources. The Interface captures multimodal user input (eg, text, audio, images) and transmits it to the Orchestrator, which processes queries using structured data transformation and decision-making based on the Perceptual Cycle Model. The Orchestrator interacts with External Sources—including healthcare data, knowledge bases, AI models, and translators—to retrieve and analyze relevant information for generating responses.

We design an LLM-powered framework with a central agent that perceives and analyzes user queries, provides appropriate responses, and manages access to external resources through Application Program Interfaces (APIs) or function calls. The user-framework interaction is bidirectional, ensuring a conversational tone for ongoing and follow-up conversations. Figure 2 shows an overview of the framework, including 3 major components: *Interface*, *Orchestrator*, and *External Sources*.

### Interface

*Interface* acts as a bridge between the users and agents, including interactive tools accessible through mobile, desktop, or web applications. It integrates multimodal communication channels, such as text and audio. The Interface receives users’ queries and subsequently transmits them to the *Orchestrator* (see Figure 2).

Within this framework, users can provide metadata (alongside their queries), including images, audio, gestures, and more. For instance, a user could capture an image of their meal and inquire about its nutritional values or calorie content, with the image serving as metadata.

### Orchestrator

The *Orchestrator* is the openCHA agent core, which is responsible for problem-solving, planning, executing actions, and providing an appropriate response based on the user query. It incorporates the concept of the Perceptual Cycle Model^32^ in openCHA, allowing it to perceive, transform, and analyze the world (ie, input query and metadata) to generate appropriate responses. To this end, the input data are aggregated, transformed into structured data, and then analyzed to plan and execute actions. Through this process, the Orchestrator interacts with external sources to acquire the required information, perform data integration and analysis, and extract insights, among other functions. In the following, we outline 5 major components of the Orchestrator.

The *Task Planner* is the LLM-enabled decision-making, planning, and reasoning core of the Orchestrator. Its primary responsibility is gathering all necessary information to answer users’ queries. To achieve this, it interprets the user’s query and metadata, identifying the necessary steps for task execution.

To transform a user query into a sequence of tasks, we incorporate the Tree of Thought^33^ prompting methods into the Task Planner. Using this prompting method, the LLM is asked to (1) generate 3 unique strategies (ie, sequences of tasks to be called with their inputs), (2) describe the pros and cons of each strategy, and (3) select one as the best strategy. An alternative prompting technique incorporated in openCHA is ReAct,^34^ which employs reasoning and action techniques to ascertain the essential tasks to be executed. openCHA offers users the flexibility to choose the prompting method that best meets the needs of their application. Other prompting techniques, such as Plan-and-Solve Prompting,^35^ could also be implemented and integrated as a *Task Planner*.

We outline the process of creating and integrating a task into openCHA in Appendix S1. We also indicate how the task is converted into an appropriate prompt, enabling the *Task Planner* to recognize the available tasks and how to invoke them. Appendix S2 provides a detailed examination of the *Task Planner’s* implementation, utilizing the Tree of Thought prompting method.

In the proposed Orchestrator, the planning part is performed in English, leveraging the superior capabilities of LLMs in this language. The framework can employ one of 2 distinct approaches if the query is in a language other than English. The first approach retrains the source language and utilizes the language model capabilities in that language to generate responses. The second approach involves translating the query into English (eg, using Google Translate), planning and executing the process in English, and translating the final answer back into the source language.

The *Task Executor* carries out actuation within the Orchestrator by following the planning and task execution steps determined by the *Task Planner*. The Task Executor has 2 primary responsibilities. First, it acts as a data converter, converting the input query and metadata and preparing it to be used by the *Task Planner*. For instance, if the question is in a language other than English, it will be translated into English using the Google Translate service.^36^ Furthermore, if the metadata contains files or images, *Task Executor* sends the metadata details to *Task Planner* for planning. Second, the *Task Executor* executes tasks generated by the *Task Planner* through interactions with external sources. The results are then relayed to the *Task Planner* to continue planning if needed. In the end, the *Task Planner* signals the end of the planning. In Appendix S2, we detail how the *Task Planner* translates planned tasks into execution instructions, enabling the *Task Executor* to properly carry out the tasks.

It is crucial to emphasize that communication between the task planner and task executor is bidirectional. An iterative process continues between the Task Executor and Task Planner until the Task Planner accumulates sufficient information to respond appropriately to the user’s inquiry. This 2-way exchange proves indispensable because, in specific scenarios, the Task Planner may necessitate intermediate information to determine subsequent actions.

The *Data Pipe* is a repository of metadata and data acquired from *External Sources* through the execution of conversational sessions. This component is essential because numerous multimodal analyses involve intermediate stages, and their associated data must be retained for future retrieval. The intermediate data might be large, surpassing token limits, or challenging to comprehend and utilize by the *Task Planner’s* or *Response Generator’s* LLM. The *Data Pipe* is automatically managed by the *Task Executor*. It monitors the stored metadata and intermediate data.

The *Data Pipe* in openCHA can range from a simple in-memory key/value storage for intermediate data to a more complex database system. The proposed framework allows developers to determine whether their tasks’ results are intermediate or should be directly returned to the LLM. Appendix S1 details how developers can configure this setting.

Additionally, we have implemented a mechanism whereby an intermediate result stored in the *Data Pipe* generates a unique key as the task’s outcome. This key is then provided in the *Task Planner* prompt, aiding the *Task Planner* in recognizing and utilizing this data as necessary. Appendix S4 illustrates sample prompts generated for tasks and demonstrates how the *Task Planner* employs the *Data Pipe* key.

The *Promptist* is responsible for transforming query text or outcomes from External Sources into suitable prompts that can be supplied to either the *Task Planner* or the *Response Generator*. The *Promptist* provides the flexibility to modify and adapt each technique, allowing for seamless integration and customization. It can be implemented using existing prompting techniques, some of which are listed as follows.

LLM-REC, proposed by Lyu et al.,^37^ employs 4 unique prompting strategies to enrich text descriptions, enhancing personalized text-based recommendations. The approach leverages the LLM to understand item characteristics, significantly improving recommendation quality. Additionally, the Hao et al.^38^ method can be leveraged, which optimizes text-to-image prompt generation through a framework called prompt adaptation. It automatically refines user inputs into model-preferred prompts. This process starts with supervised fine-tuning of a pretrained language model using a curated set of prompts. It then employs reinforcement learning, guided by a reward function, to identify more effective prompts that produce aesthetically pleasing images aligned with user intentions. Furthermore, the instructions provided by OpenAI on creating more effective prompts can be used.^39^

The *Response Generator* is an LLM-based module responsible for preparing the response. It refines the gathered information by the *Task Planner*, converting it into an understandable format and inferring the appropriate response. We separate the *Response Generator* and *Task Planner* to allow flexibility in choosing diverse LLM models and prompting techniques for these components. This division ensures that the *Task Planner* focuses solely on planning without responding to users, while the *Response Generator* utilizes gathered information to deliver conclusive responses. This segregation facilitates the *Response Generator* in addressing aspects of *empathy* and *companionship* in conversations. In contrast, the *Task Planner* primarily handles *personalization* and the *up-to-dateness* of conversations. Appendix S3 outlines the implementation of the *Response Generator* and how it utilizes results collected by the *Task Planner* to respond to the user effectively.

### External sources

*External Sources* play a pivotal role in obtaining essential information from the broader world. Typically, these External Sources furnish APIs that the Orchestrator can use to retrieve required data, process them using AI or analysis tools, and extract meaningful health information. In openCHA, we integrate with 4 primary external sources, which we found critical for CHAs (see Figure 2).

*Healthcare Data Source* enables the collection, ingestion, and integration of data captured from a variety of sources, such as Electronic Health Record (EHR), smartphones, and smartwatches, for healthcare purposes.^40^ Examples of data sources are mHealth platforms and healthcare databases. mHealth platforms have garnered significant attention in the recent wave of healthcare digitalization, enabling ubiquitous health monitoring.^41^^,^^42^ The data encompass various modalities, including biosignals (eg, PPG collected via a smartwatch), images (eg, captured via user’s smartphone), videos, tabular data (eg, demographic data gathered from EHR), and more. Notable examples of such healthcare platforms include ZotCare^40^ and ilumivu,^43^ offering APIs for third-party integration. In our context, the Orchestrator functions as a third party, accessing user data with their consent.

*Knowledge Base* fetches the most current and pertinent data from healthcare sources, such as healthcare literature, reputable websites, or knowledge graphs using search engines or retrieval models.^44–47^ Accessing this retrieved information equips CHAs with up-to-date, personalized knowledge, enhancing its trustworthiness while reducing hallucination and bias. openCHA allows the integration of various knowledge bases to be defined and configured as tasks.

*AI and Analysis Models* provide data analytics tools to extract information, associations, and insights from data,^19^^,^^48^ playing a crucial role in the evolving landscape of LLM-healthcare integration, enhancing trustworthiness and personalization. They can perform various tasks, including data denoising, abstraction, classification, and event detection, to mention a few.^19^^,^^48^ As generative models, LLMs cannot effectively perform extensive computations or act as machine learning inferences on data. The AI platforms empower our framework to leverage existing health data analytic approaches.

*Translators* effectively convert various languages into widely spoken languages, such as English, thereby enhancing the accessibility and inclusivity of CHAs. Existing agents face limitations that hinder their usability for large communities globally. Universal text literacy for CHAs often narrows their reach and positions them as a privilege.^49–51^ Many underserved communities face obstacles while using CHAs due to their educational disparities, financial constraints, and biases that favor developed nations within existing technological paradigms. Our framework integrates with Translator platforms and is designed to accommodate and support communication with diverse communities. This integration enhances the overall usability of CHAs.

The selection of the external sources is based on the information and knowledge they provide and their interaction with the Orchestrator. The Orchestrator handles various data types in the proposed framework, including text, JSON formatted data, or unstructured data such as images and audio. This design ensures that any external source capable of returning results in these formats is supported.

## Demonstration

**Figure 3.**
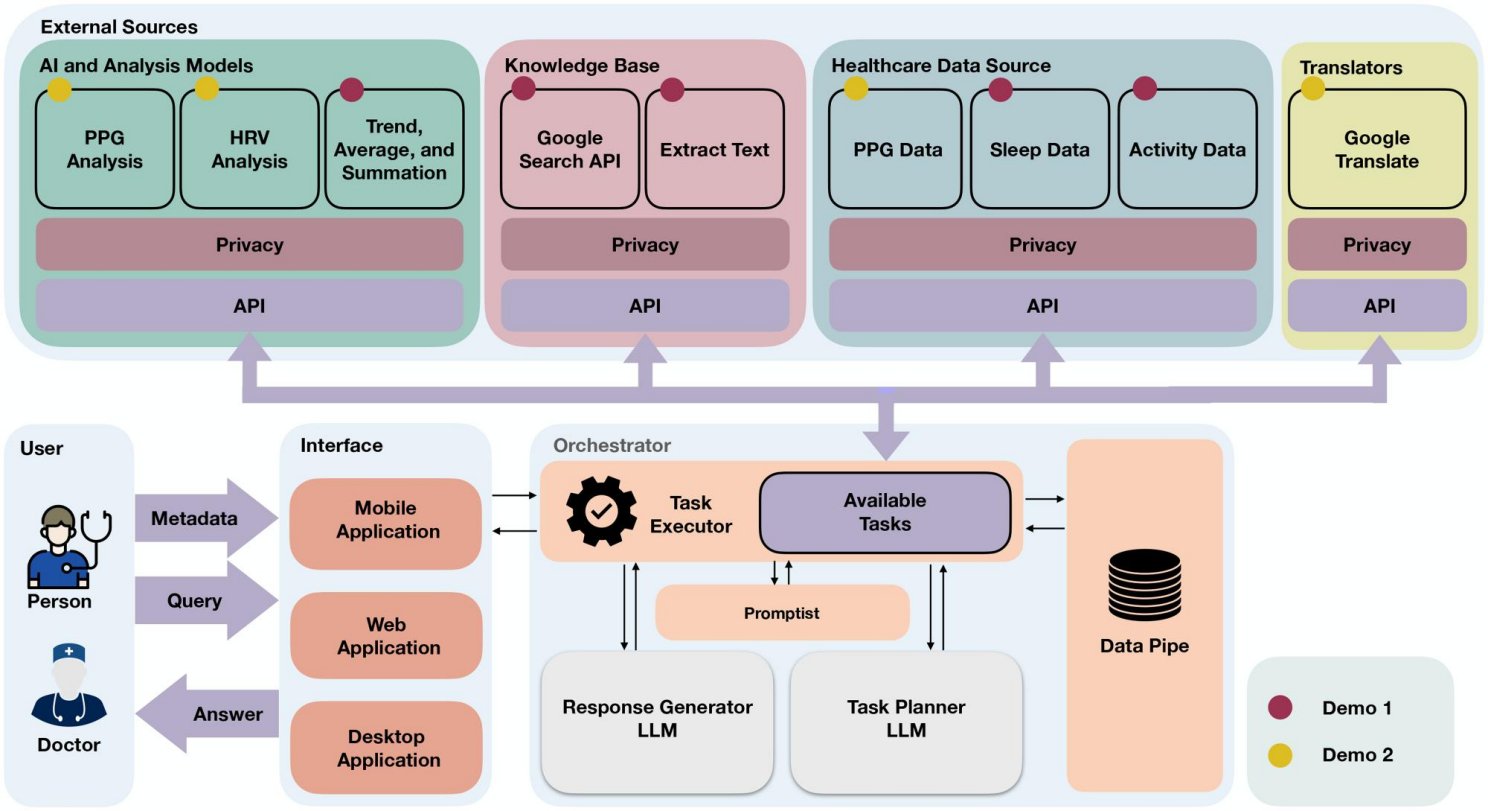
Overview of tasks and components used in two openCHA demos. Demo 1 (red dots) retrieves and analyzes sleep and activity data from a health database. Demo 2 (yellow dots) processes wearable PPG signals to estimate stress using HRV features and an AI model.

We demonstrate the capabilities of openCHA through 2 distinct demos. In these demos, 2 different CHAs are built and configured. These demonstrations highlight how, in the final CHA, LLMs’ planning and reasoning abilities can comprehend user queries and translate them into appropriate task executions. Each demonstration involves linking a set of implemented tasks to openCHA. We then showcase how openCHA planning effectively sequences and executes tasks, ensuring they are performed in the correct order with the right inputs. After executing all necessary tasks, the results are sent to the Response Generator to provide the final response. The overview of the implemented tasks and their utilization in each demo is depicted in Figure 3.

### Demo 1: Patient health record reporting

The first demo showcases a CHA built using openCHA to interact with patient data stored in a database, conduct data analysis, generate health reports upon request, and ask follow-up questions. Examples of the user’s questions could be: “Provide a sleep summary of Patient 5 during August 2020,” “Is Patient 5 REM sleep enough during August 2020?,” “How much is the total step count of Patient 5 during August 2020?” or “Provide an activity summary of Patient 5 during 2020.” For this demo, we implement 2 tasks for retrieving sleep and physical activity from a health monitoring dataset.^52^ The data utilized in this demo is a part of an extensive longitudinal study focusing on the mental health of college students, as documented in.^52^ Moreover, we develop analytical tasks capable of executing basic statistical analysis (eg, computing trends and averaging). Finally, we also add Google Search and Extract Text tasks so that the current setup of openCHA can access internet information. The involved tasks in this demo are marked by red dots in Figure 3.

**Figure 4.**
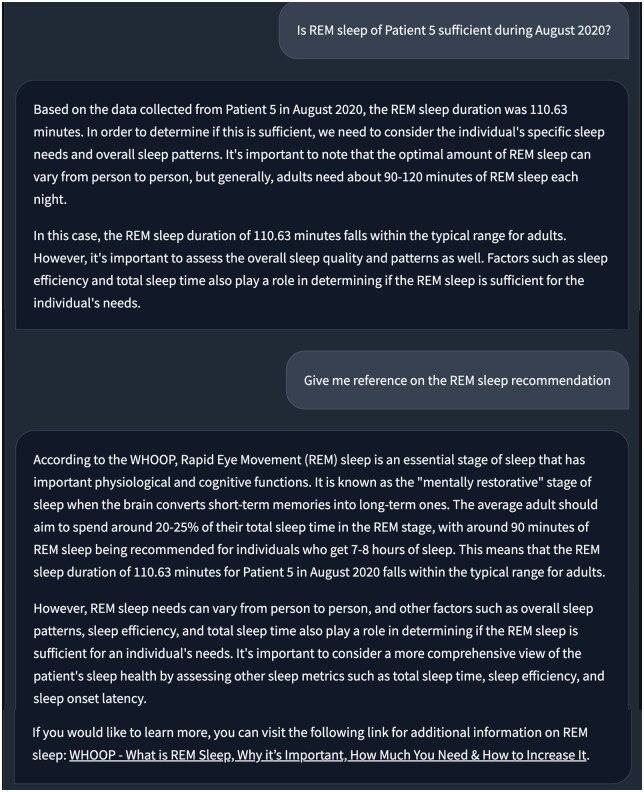
Demo 1. Patient health record reporting and follow up.

Figure 4 illustrates sample queries and the corresponding responses from openCHA. The Task Planner identifies the correct tasks, their execution order, and the appropriate inputs to be used. Table 2 showcases the strategy chosen by the Task Planner using the Tree of Thought prompting technique.

**Table 2.** The strategies suggested by Tree of Thought prompting technique for the first question.

Decision	I will proceed to directly analyze the REM sleep data of Patient 5 for August 2020, providing a precise and specific conclusion. Execution: 1. Use the sleep_get tool to obtain the REM sleep data for Patient 5 in August 2020. 2. Utilize the sleep_analysis tool to analyze the REM sleep duration and efficiency for August 2020.

### Demo 2: Objective stress level estimation with multilanguage interaction

Demo 2 showcases a CHA built using openCHA to conduct signal processing and objective stress level estimation. We show that the configured CHA can answer the query in multiple languages. This is achieved by interacting with a translator, health data sources, and AI models. Examples of interactions include inquiries such as “Retrieve the stress level of Patient 5 on August 29th, 2020” and “What is the average heart rate of Patient 5 during August 2020?” To fulfill our objective, we implemented 3 distinct tasks (yellow dots in Figure 3). The first task involved acquiring Photoplethysmogram (PPG) data from the patients. PPG data were gathered using Samsung Gear Sport smartwatches,^53^ with a sampling frequency of 20 Hz, while participants were in free-living conditions. The data is part of the.^52^ The second task performs PPG signal processing to extract heart rate variability (HRV) metrics. For this purpose, we utilize the Neurokit^54^ Python library. In our case study, we extract a total of 32 HRV parameters, including metrics such as the root mean square of successive differences between normal heartbeats (RMSSD), low-frequency (LF), and high-frequency (HF) values.^55^^,^^56^ The third task estimates stress levels based on HRV using an AI model. Initially, we employed an autoencoder to reduce the dataset’s 32 HRV features to 12. Subsequently, a 4-layer neural network categorizes the 12 features into 5 stress levels. The evaluation of the stress estimation model demonstrates an 86% accuracy rate on a test set.

**Figure 5.**
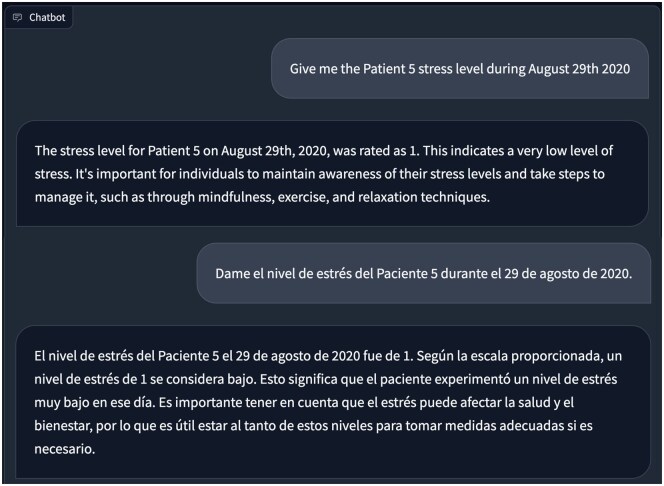
Demo 2. Objective stress level estimation. The question is asked in English and Spanish.

Figure 5 depicts example queries and the corresponding responses from openCHA. The Task Planner’s approach involves initially retrieving the PPG data of Patient 5 on August 29, 2020. Subsequently, the obtained result is forwarded to the PPG analysis task to extract HRV metrics. Lastly, the planner initiates the execution of stress analysis tasks, providing the HRV metrics for this task. Table 3 displays the chosen strategy by the Task Planner utilizing the Tree of Thought prompting technique. The ultimate estimated stress, along with an explanation, is then returned to the user. Table 3 indicates when the user inquired, “Give me the Patient 5 stress level during August 29th 2020,” the Tree of Thought Planner successfully identified the relationship between the patient’s PPG signal and the HRV feature. Subsequently, the CHA utilized this information in the *stress_analysis* tool to determine the stress level. Importantly, none of this information was pre-supplied to the planner, which discerned this connection independently through the Tree of Thought process.

**Table 3.** The strategies suggested by Tree of Thought prompting technique for the first question.

Decision	The best strategy provides both detailed PPG analysis and an estimation of the stress level, which offers a comprehensive view of the patient’s health status. Execution: 1. Use ppg_get tool to retrieve the PPG data for Patient 5 during August 29th, 2020. 2. Analyze the PPG data with ppg_analysis tool to obtain the heart rate. 3. Use stress_analysis to estimate the stress level based on the obtained PPG data.

### openCHA use cases

To indicate the usability of openCHA across various applications, we outline several use cases that have utilized the framework to build their customized CHA for their research as follows.

1. ChatDiet,^57^ utilizing openCHA as its core architecture, introduced a personalized, nutrition-oriented food recommendation agent that seamlessly integrates personal and population models. Leveraging these external sources, ChatDiet delivers tailored food suggestions, enhancing traditional food recommendation services with dynamic, personalized, and explainable recommendations. This CHA connects nutritional values to specific health outcomes, such as sleep quality, adeptly finding data-driven connections between individual health data and broader population trends. In scenarios requiring dietary substitutions, ChatDiet identifies closely matching food items, maintaining nutritional continuity and personalization.
    In a comprehensive case study, ChatDiet demonstrated its proficiency by achieving a 92% effectiveness rate, significantly outperforming established solutions like ChatGPT. The ChatDiet performance underscores its ability to handle the complexities of personalized nutrition, making informed adjustments and suggestions that are both scientifically grounded and highly customized to individual health profiles.
2. Knowledge-infused LLM-powered CHA for diabetic patients^58^ is developed by integrating domain-specific knowledge and analytical tools as external sources using openCHA. This integration included incorporating American Diabetes Association dietary guidelines and deploying analytical tools for nutritional intake calculation, resulting in superior performance compared to GPT4 in managing diabetes through tailored dietary recommendations, as demonstrated by an evaluation of 100 diabetes-related questions.
3. openCHA was employed to develop an agent for evaluating the safety and reliability of mental health chatbots.^59^ This agent’s evaluation capabilities were compared with expert assessments and several existing LLMs, including GPT4, Claude, Gemini, and Mistral. Guidelines and benchmarks introduced by experts and Internet search served as external sources linked to openCHA. The agent demonstrated superior accuracy, achieving the lowest mean absolute error (MAE) against experts’ scores—a reduction by a factor of 1 compared to LLMs’ scores, with the maximum MAE being 10—and provided unbiased evaluation scores.
4. The Empathy-enhanced CHA^60^ was developed to interpret and respond to users’ emotional states through multimodal dialogue, representing a significant step forward in providing contextually aware and empathetically resonant support in the mental health field. This paper utilized speech-to-text, text-to-speech, and speech emotion detection models as external sources connected to openCHA. The created CHA’s robustness is tested by conducting 500 tests, randomly selecting questions infused with 1 of 3 emotions. Performance metrics revealed an accuracy of 89% in accurately identifying emotional states and retrieving pertinent information, and 61% in using these emotions to perform relevant Internet searches.
5. An LLM-Powered Agent for Physiological Data Analysis: A Case Study on PPG-based Heart Rate Estimation^61^ was developed using the openCHA framework to better integrate LLMs with traditional analytical tools for health insights. This agent employs an orchestration layer that synergizes user interactions, data inputs, and analytical methods to generate precise cardiovascular health assessments from PPG signals. For validation, a dataset involving home-based sleep monitoring of 45 individuals was used, assessing parameters such as Total Sleep Time, Sleep Efficiency, and Wake After Sleep Onset. The performance comparison, specifically the MAE results, are detailed in Table 4. These results, validated against established benchmarks like OpenAI GPT-4o-mini and GPT-4o with ECG as the gold standard, demonstrated significantly enhanced accuracy and reliability in heart rate estimation.

**Table 4.** Summary of evaluation results from two use cases. Bold values indicate the performance of the proposed openCHA agent system across various metrics.

Knowledge-infused LLM-powered CHA^58^						
	**GPT4**			**Proposed CHA**		

**Accuracy**	51.8%			**92.1%**		

**Evaluator Agent^59^**						

	**GPT4**	**Misteral**	**Claude3-Opus**	**Gemini**	**Embeddings**	**CHA Evaluator**

**MAE**	2.29	1.43	1.79	2.03	0.63	**0.31**

***P*-value**	7.30e−23	8.55e−13	2.72e−15	1.85e−18	1.07e−07	**0.1961**

**LLM-Powered Agent for Physiological Data Analysis^61^**						

	**GPT-4o-Mini**		**GPT-4o**		**Proposed Agent**	

**MAE**	9.41		8.93		**2.83**	

Table 4 provides a comparative analysis of the evaluation metrics for use cases numbers 2, 3, and 5, detailing the performance differences between the implemented CHAs and other LLMs. In the “Knowledge-Infused LLM-powered CHA for Diabetic Patients” study, the CHA demonstrated a remarkable 92% accuracy in assessing daily nutrient intake risks, significantly outperforming GPT4, whose accuracy was close to random. The “Evaluator Agent CHA” use case compared the CHA evaluator with various models, achieving the lowest MAE relative to expert scores and showing statistically significant results that highlighted its reduced bias compared to other methods. Additionally, in the “LLM-Powered Agent for Physiological Data Analysis,” the MAE for heart rate estimation from PPG signals was calculated against 2 GPT models, with the proposed agent displaying the lowest MAE, indicating superior accuracy.

It should be noted that the accuracy and other performance metrics for CHAs developed with openCHA are influenced by various configurations and choices made during the development process. For more information on evaluation methods and metrics, please refer to the “Accuracy and Evaluation” section in “Discussion.”

To see more demonstrations on how the openCHA works in real setup, we have uploaded multiple YouTube videos (https://www.youtube.com/watch?v=w48sPlF5zhs, https://www.youtube.com/watch?v=PWxL_OgWGfE&t=3s, https://www.youtube.com/watch?v=c-7IEBaRSyQ, and https://www.youtube.com/watch?v=rHXpk_P5n6Y).

## Discussion

### openCHA potentials and limitations

In this section, we briefly discuss our proposed framework’s capabilities, potentials, and limitations.

#### Flexibility

openCHA provides a high level of *flexibility* to integrate LLMs with external data sources, knowledge bases, and analytical tools. The proposed components can be developed and replaced according to the requirements of the healthcare application in question. For instance, new external sources can be effortlessly integrated and introduced as new tasks into openCHA. The LLMs employed in openCHA can be readily swapped with fine-tuned or more healthcare-specific LLMs. Similarly, the Planner prompting technique and decision-making processes are modifiable. This flexibility facilitates collaboration among diverse research communities, enabling them to contribute to various aspects of CHAs. Appendix S1 shows how a new task can be defined and introduced into openCHA.

#### Explainability

**Figure 6.**
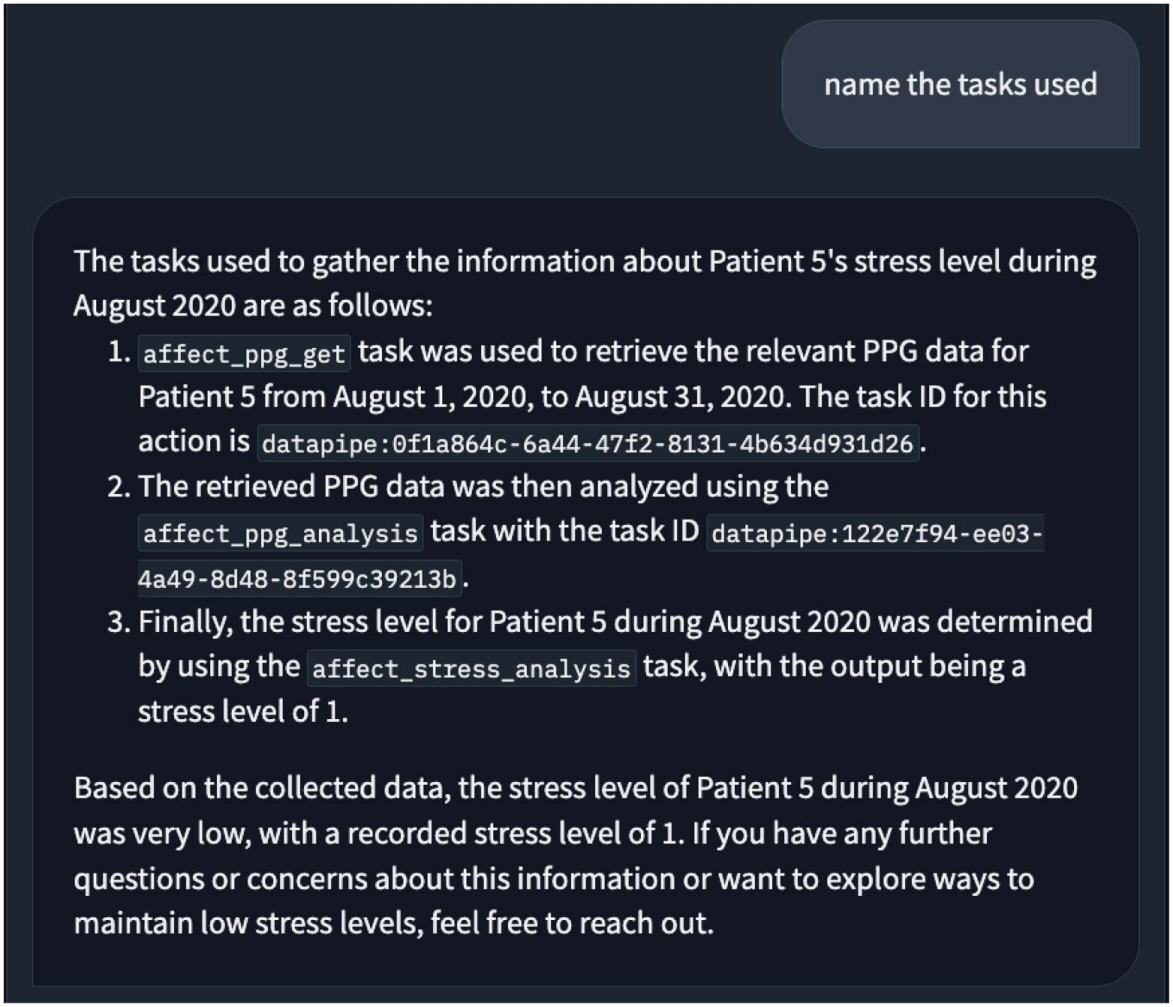
An example screenshot indicating how openCHA improves explainability in conversations.

openCHA enhances *explainability* for CHAs, allowing users to inquire about the tools and actions used to generate a response. As detailed in Appendices S2 and S3, openCHA maintains a “previous actions” section that records past conversations and tasks. When queried about task usage, it lists the executed tasks and their applications, enhancing transparency and fostering trust between users and CHAs. For instance, in Demo 2, when a user asks, “Name the tasks used,” openCHA responds by detailing that PPG and HRV data were utilized to determine stress levels. An example of this interaction is shown in Figure 6.

#### Personalization

The openCHA framework enhances personalization by allowing the integration of individuals’ information and analytics tools from healthcare systems or local databases as external sources. The quality of these external sources greatly influences the effectiveness of the personalization. For example, ChatDiet^57^ utilizes personal dietary preferences and population data, along with an analysis of nutrients’ effects on health outcomes like sleep quality, to enhance its food recommendations significantly. This strategy not only heightens the accuracy of the recommendations but also ensures they are precisely tailored to meet individual dietary needs.

#### Reliability

openCHA boosts the reliability of answers by leveraging validated information and computations as external sources. Our framework is tailored to effectively utilize existing LLMs for complex healthcare tasks, strategically offloading computational and sensitive information tasks to external sources while reserving LLMs primarily for reasoning and generating responses. For instance, the paper “Knowledge-infused LLM-powered CHA for diabetic patients”^58^ demonstrates the benefits of integrating external knowledge to accurately determine nutritional values and align them with established guidelines, highlighting inaccuracies in nutritional estimations when solely relying on GPT4 LLM for data access and calculations.

#### Latency

Utilizing multiple external sources offers benefits, but it can also affect the model’s response time, potentially leading to increased latency in the CHA. As the number of tasks and steps within the framework expands, there could be a rise in response time, which might diminish usability. Recent research studies^62–64^ explore new methods for executing tasks in parallel. LLM-Tool Compiler^62^ enhances LLM by fusing similar API calls into a single function, improving parallelization and reducing system latency and costs. LLMCompiler^63^ enhances LLMs by enabling parallel execution of function calls, improving efficiency with 3 core components: a Function Calling Planner, a Task Fetching Unit, and an Executor. This approach significantly reduces latency, cuts costs, and boosts accuracy by optimizing function call orchestration, applicable to both open and closed-source models. AsyncLM^64^ introduces a method to improve the efficiency of LLMs by enabling asynchronous function calling, allowing concurrent execution of multiple function calls without waiting for each to complete. This system utilizes an interrupt mechanism to notify the LLM in real-time as function calls return, significantly reducing task completion latency. Researchers can integrate these methods into openCHA’s Orchestrator to facilitate task parallelization and decrease latency.

#### Token limit

Token limits in LLMs present a challenge for accommodating tasks within the Task Planner. However, recent advancements^65^ indicate progress in extending LLM token limits, which helps mitigate this issue. Researchers can employ new LLMs with expanded token limits within openCHA to address this issue.

#### Privacy and security

Privacy and security are paramount in the development of CHAs using the openCHA framework, especially for healthcare applications that handle sensitive user data. Strong privacy measures are essential to mitigate risks such as unauthorized access, data breaches, and identity theft, which can have severe consequences.^66^^,^^67^ Although openCHA provides foundational tools for building CHAs, it does not oversee the deployment of these agents. It is important to note that compliance with privacy regulations like HIPAA and GDPR depends on the specific implementation by developers and researchers. This includes maintaining privacy at multiple deployment levels and verifying the security and HIPAA compliance of any external sources integrated into the CHA. Measures to ensure data confidentiality include granting limited access to CHAs with user permission and employing de-identification and anonymization techniques.^68^ Additionally, it is critical to prevent the use of user-provided data for training or fine-tuning LLMs to avoid storing sensitive information.

#### Scalability

The scalability of each CHA and its deployment is determined by how developers and researchers configure and adapt the CHA to their specific needs. OpenCHA is primarily a research-focused framework that provides foundational tools for building CHAs, but it does not manage the deployment or scalability of these agents.

### Study limitations and future work

In this section, we outline the study limitations and future research directions.

#### Planning robustness

Since we utilize LLMs for planning and response generation, there is still the inherent risk of biases or trustfulness issues. Our framework aims to enhance the robustness of planning by integrating external sources to reduce these problems, though it cannot ensure their complete elimination. To enhance the planning robustness, we will explore using agentic design patterns like the self-consistency^69^ method or new reasoning techniques.

#### Accuracy and evaluation

Accuracy and evaluation in our framework hinge on the configuration choices made by researchers, such as the selected external sources, LLM, and planning technique. Since knowledge, data, and analytics are outsourced to external sources, the quality of these sources plays a crucial role in enhancing accuracy; better external sources increase the likelihood of achieving superior results.

Two distinct assessments are necessary to evaluate such systems. The first evaluates the accuracy of external sources, whether they are AI models or knowledge bases. The second assesses the overall configured and constructed CHA to determine if it behaves as expected. Several metrics and evaluation methods are recommended in,^70^ with additional evaluation techniques explored in.^57–60^ These evaluations are application-specific, and our framework provides extensive customization capabilities to suit different use cases and requirements. In our future work, we will explore more evaluation techniques.

#### User query ambiguity

Understanding user intentions presents a significant challenge due to query ambiguity, often caused by vague or incomplete information and a lack of necessary external sources connected to openCHA for the specific application. To enhance response accuracy, our future work involves refining openCHA’s ability to clarify user intentions. Key strategies include employing targeted follow-up questions, improving comprehension of the user’s situation, and the precision of responses.

## Conclusion

We demonstrated openCHA’s ability to develop CHAs across multiple health domains using 2 demos and 5 use cases, showcasing its effectiveness in various complex healthcare tasks. The developed CHAs achieved notable results: In diabetic patient management, the developed CHA achieved a 92.1% accuracy, surpassing GPT4’s 51.8%. In food recommendations, the developed CHA outperformed GPT4. The CHA also excelled as an evaluator for mental health chatbots, recording the lowest MAE at 0.31, compared to competitors like GPT, Misteral, Gemini, and Claude. Additionally, the empathy-enabled CHA identified emotional states with 89% accuracy, and in physiological data analysis of heart rate from PPG signals, the developed CHA achieved an MAE of 2.83, far lower than GPT-4o’s 8.93. These outcomes illustrated that CHAs built with openCHA exceeded LLM-based methods’ performance by leveraging capabilities for explainability, personalization, and reliability, thus pushing the boundaries of current healthcare technology solutions.

## Supplementary Material

ooaf067_Supplementary_Data

## Data Availability

No original data were generated, and no original or third-party data were analyzed in support of this research.
