# Supplementary material for: Conversational health agents: a personalized large language model-powered agent framework
Source: JAMIA Open. 2025 Jul 6;8(4):ooaf067. doi: 10.1093/jamiaopen/ooaf067 (PMC12228965; doi:10.1093/jamiaopen/ooaf067)
Supplement: ooaf067_Supplementary_Data [file ooaf067_supplementary_data.pdf]

APPENDIX 1 - SAMPLE TASK IMPLEMENTATION

We encourage readers and openCHA users to review our documentation page [1] for the latest updates on openCHA.

In this appendix, we will detail the curation of tasks within the openCHA framework and their introduction as external sources to the Task Planner. Initially, we will describe the types of tasks that can be added to the framework. Subsequently, we will outline the implementation process for tasks within the framework. Finally, we will present an example of a task that has already been implemented, along with sample prompts associated with it.

Tasks may be implemented locally or as a service. Local implementation entails coding all necessary components on-site to ensure successful task execution, which may involve providing a Python library, a GitHub repository, or a complete task implementation.

Service-based implementation involves hosting services on a server and offering APIs for utilization. In this scenario, the task should invoke these APIs using Python libraries. It is incumbent upon the service provider to maintain the task and supply comprehensive documentation for users, including registration procedures, API key acquisition, and privacy policies.

Figure 1 displays an example of a task implementation in openCHA designed to perform a Google search and return the first search result URL. Each task includes configurations to aid the Task Planner LLM in under-

[1] `https://docs.opencha.com/index.html`

```python
class GoogleSearch(BaseTask):
    """
    **Description:**

        This task uses google search to search the query in the internet and returns the urls.

    """

    name: str = "google_search"
    chat_name: str = "GoogleSearch"
    description: str = "Uses google to search the internet for the requested query and returns the url of the top website."
    dependencies: List[str] = []
    inputs: List[str] = ["It should be a search query."]
    outputs: List[str] = [
        "It returns a json object containing key: **url**. For example: {'url': 'http://google.com'}"
    ]
    output_type: bool = False
    search_engine: Any = None

    @model_validator(mode="before")
    def validate_environment(cls, values: Dict) -> Dict:
        """
            Validate that api key and python package exists in environment.

        Args:
            values (Dict): The dictionary of attribute values.
        Return:
            Dict: The updated dictionary of attribute values.
        Raise:
            ValueError: If the SerpAPI python package is not installed.

        """

        try:
            from googlesearch import search

            values["search_engine"] = search
        except ImportError:
            raise ValueError(
                "Could not import googlesearch python package. "
                "Please install it with `pip install googlesearch-python`."
            )
        return values

    def _execute(
        self,
        inputs: List[Any] = None,
    ) -> str:
        query = inputs[0]
        result = {"url": list(self.search_engine(query))[0]}
        return result

    def explain(
        self,
    ) -> str:
        return "This task uses google search to search the query in the internet and returns the urls."
```

Figure 1: A sample implemented task inside openCHA framework. Each task includes prompt parameters which helps our Task Planner LLM understand what types of data are needed and how to provide them to the current task.

standing when and how to use this task. The parameters are as follows:

name

Purpose: Uniquely identifies the task within the system, and this name will appear in the interface for task selection.

Convention: Generally in lowercase underscore_case, often derived from the task's functionality for better readability and maintainability.

Example: name = "google_search"

chat_name:

Purpose: Used for referencing the task in user interfaces or chats, particularly for explainability. If a user inquires about the tasks used, these names will be displayed.

Convention: Typically in CamelCase, should be descriptive yet concise.

Example: chat_name = "GoogleSearch"

description:

Purpose: Provides an explanation of the task's function. This information helps the Task Planner LLM decide when to deploy the task.

Convention: The description should be comprehensive, outlining task capabilities, prioritization, or specific conditions for its use. For instance, ensuring the Task Planner prioritizes patient data analysis over frequent internet searches can be mentioned here with a low priority setting.

Example: description = "Uses Google to search the internet for the requested query and returns the URL of the top website."

dependencies:

Purpose: Lists other tasks or services this task depends on, informing the Task Planner LLM of these dependencies during planning.

Convention: An array of task identifiers that match the name attribute of dependent tasks.

Example: dependencies = [TaskType.FETCH_WEATHER_DATA]

inputs:

Purpose: Specifies the inputs required by the task. This parameter is crucial as it guides the Task Planner LLM to provide the appropriate inputs for this task's "_execute" function.

Convention: An array of strings, each describing a specific input.

Example: inputs = ["It should be a search query."]

outputs:

Purpose: Details what the task returns, assisting the Task Planner LLM in understanding the return structure for proper handling of the output.

Convention: An array of strings describing each output, with a full description of the return data format.

Example: outputs = ["It returns a JSON object containing key: url. For example: 'url': 'http://google.com'"]

output_type

Purpose: Instructs the Orchestrator on how to handle the output of this task. If output_type is True, the data is stored in the Datapipe; otherwise, it is directly returned to the Task Planner LLM. This flexibility allows task designers to determine whether their task involves intermediate information

for use by other tasks or provides the final answer.

Convention: Boolean values (True or False).

_execute function

Purpose: This function must be implemented correctly. If all other parameters are set accurately, developers can trust that the Task Planner LLM will invoke this function with the appropriate inputs. For instance, as shown in Figure 1, the sole expected input is a search query. The Task Planner LLM will correctly supply the search query as an input to the "_execute" function, accessible as the first element in the inputs array (since the only entry in the "inputs" configuration pertains to the search query, this array will contain just one item). Utilizing the google_engine Python library, this function searches for the query online and returns the URL. Note that the result we are returning for the Google search task aligns with what is specified in the "outputs" parameter, demonstrating the coherence between the task setup and its execution.

APPENDIX 2 - TASK PLANNER SAMPLE IMPLEMENTATION

We encourage readers and openCHA users to review our documentation page [2] for the latest updates on openCHA.

In this appendix, we elaborate on the implementation of the Tree of Thought prompting technique. We describe how available tasks are introduced to the Tree of Thought, the process of curating and generating the

[2] `https://docs.opencha.com/index.html`

planning prompt, and ultimately, how the final planning results are transformed into executable tasks by the Task Executor.

The core component of the Task Planner is the selection of an appropriate planning technique. Among various techniques using LLMs that have proven practical, we opted for the Tree of Thought approach.

To effectively implement the Tree of Thought, we structured the planning process into two main sections. Initially, we prepare a comprehensive prompt that includes a list of available tasks, relevant metadata, records of previously performed tasks, conversation history, and user input. This setup ensures the planner is thoroughly informed about the tasks it can call upon, the metadata that should be used or passed along, and the context of past interactions to prevent redundant planning and maintain the continuity of conversations.

Figure 2 illustrates the Tree of Thought planning prompt. In this phase, the Tree of Thought uses all this gathered information and the user's query to devise three distinct task sequences or strategies. Each strategy is designed to collect the necessary information to address the query efficiently. Next, it is asked to provide the pros and cons of each strategy, ultimately selecting the most suitable one as the final decision.

In the second stage of the planning process, our objective is to translate the chosen decision into sequences of task functions that the Orchestrator can understand and execute. Within the Orchestrator, we have implemented a function named execute_task, which serves as an interface to retrieve and execute tasks with the appropriate inputs accurately.

```
          """As a knowledgeable and empathetic health assistant, your primary objective is to provide the user with precise and valuable \
information regarding their health and well-being. Utilize the available tools effectively to answer health-related queries. \
Here are the tools at your disposal:
{tool_names}

The following is the format of the information provided:
MetaData: this contains the name of data files of different types like image, audio, video, and text. You can pass these files to tools when needed.
History: the history of previous chats happened. Review the history for any previous responses relevant to the current query
PreviousActions: the list of already performed actions. You should start planning knowing that these actions are performed.
Question: the input question you must answer.

Considering previously actions and their results, use the tools and provided information, first suggest three \
creative strategies with detailed explanation consisting of sequences of tools to properly answer the user query. \
Make sure the strategies are comprehensive enough and use proper tools. The tools constraints should be always satisfied. \

After specifying the strategies, mention the pros and cons of each strategy. \
In the end decide the best strategy and write the detailed tool executions step by step.
start your final decision with
'Decision:'.

Begin!

MetaData:
{meta}
========================
{previous_actions}
========================
{history}
========================
USER: {input} \n CHA:
```

Figure 2: The first stage Tree of Thought planning prompt

Our second prompt instructs the LLM to invoke this execute_task function, ensuring that the correct inputs are provided for each task. Figure 3 displays this second prompt, showcasing how we structure these commands within the LLM to streamline task execution.

APPENDIX 3 - RESPONSE GENERATOR IMPLEMENTATION

We encourage readers and openCHA users to review our documentation page [3] for the latest updates on openCHA.

In this appendix, we provide a detailed explanation of the Response Generator's implementation. We begin by distinguishing between the Response Generator and the Task Planner. Following this, we demonstrate how the

[3] https://docs.opencha.com/index.html

````
            """
{strategy}
=========================
{previous_actions}
=========================
Tools:
{tool_names}
=========================

You are skilled python programmer that can solve problems and convert them into python codes. \
Using the selected final strategy mentioned in the 'Decision:
', create a python code inside a ```python ``` block that outlines a sequence of steps using the Tools. \
assume that there is an **self.execute_task** function that can execute the tools in it. The execute_task \
recieves task name and an array of the inputs and returns the result. Make sure that you always pass and array as second argument. \
You can call tools like this: \
**task_result = self.execute_task('tool_name', ['input_1', 'input2', ...])**. \
The flow should utilize this style representing the tools available. Make sure all the execute_task calls outputs are stored in a variable.\
If a step's output is required as input for a subsequent step, ensure the python code captures this dependency clearly. \
The output variables should directly passed as inputs with no changes in the wording.
If the tool input is a datapipe only put the variable as the input. \
For each tool, include necessary parameters directly without any names and assume each will return an output. \
The outputs' description are provided for each Tool individually. Make sure you use the directives when passing the outputs.

Question: {input}
"""
````

Figure 3: The second stage Tree of Thought planning prompt

outcomes of planning and task execution are delivered to the Response Generator. Lastly, we present the prompts employed to ensure that the Response Generator depends on the tasks' responses rather than the internal knowledge of the LLM used for generating responses.

One of the key objectives of the Agentic approach is to create a comprehensive mega prompt that allows an LLM to generate more precise answers by incorporating all necessary information. Our innovation centers on dynamically generating this mega prompt in response to user queries. The Task Planner initiates this process by determining the sequence of tasks it deems necessary to accurately address the current question.

Once planning is complete, the Orchestrator compiles the results of these tasks to create the mega prompt. The composition of this prompt is influenced by the nature of the tasks and their outputs. For instance, if a task's

```
"===========Thinker: {thinker}==========\n\n"
"System: {prefix}. You are very helpful empathetic health assistant and your goal is to help the user to get accurate information about "
"his/her health and well-being, Using the Thinker gathered information and the History, Provide a empathetic proper answer to the user. "
"Consider Thinker as your trusted source and use whatever is provided by it."
"Make sure that the answer is explanatory enough without repeatition"
"Don't change Thinker returned urls or references. "
"Also add explanations based on instructions from the "
"Thinker don't directly put the instructions in the final answer to the user."
"Never answer outside of the Thinker's provided information."
"Additionally, refrain from including or using any keys, such as 'datapipe:6d808840-1fbe-45a5-859a-abfbfee93d0e,' in your final response."
"Return all `address:[path]` exactly as they are."
"User: {query}"
```

Figure 4: The Response Generator prompt.

output_type is set to true, indicating that the result should be stored in the Datapipe for subsequent analysis task use, the Orchestrator will omit this result from the prompt. The focus in this case is on the analysis's outcome rather than intermediate results. Similarly, for tasks that produce metadata, such as an image, the metadata itself isn't passed directly to the Response Generator. Instead, descriptive information about the metadata, like details of a plot image and relevant data insights, are provided to the Response Generator, indicating that this information accompanies the image.

Figure 4 illustrates the prompt provided to the Response Generator LLM. Initially, the Orchestrator formats answers derived from the task results, which we refer to as the "thinker" part of the prompt. The LLM is then instructed to rely exclusively on this thinker prompt, disregarding its internal knowledge. Additionally, we include guidelines to help the LLM understand specific system implementations, such as Datapipe access keys or metadata details.

In the openCHA framework, this prompt is customizable. A "prefix" feature allows developers to add information to better align the response

generator with their specific task requirements.

APPENDIX 4 - SAMPLE PROMPTS

In this Appendix, we demonstrate the generated prompt for a simple example involving two tasks: Internet search and webpage information extraction. The user's query, "How to improve my sleep," is first directed to the Task Planner. Table 1 displays the initial planning stage where the Tree of Thought Planner is requested to develop three strategies, evaluate their pros and cons, and choose one to execute. Table 2 presents the prompt for the second planning stage, where the LLM is instructed to generate Python code that the Orchestrator can execute based on the chosen strategy, along with the necessary inputs. Table 3 and 4 provides an example of the generated code, illustrating how the LLM correctly inputs the query "tips to improve sleep" for the google_search task. Table 5 shows how openCHA creates a prompt to assist the Response Generator in utilizing the tasks' results to respond to the user's query effectively.

Additionally, Table 6 provides an example of a prompt for a task that has multiple inputs and outputs, with the result being stored in the Data Pipe. Table 7 illustrates the process of passing the Data Pipe key between tasks. In this setup, each task returns a Data Pipe key, and within the generated code, the output from one task is appropriately passed to the next task as its input. The execute_task function manages access to the Data Pipe when a key is provided, ensuring that the results of the tasks are not directly transmitted

to the LLM.

Table 1: Tree of Thought Planning first planning stage.

As a knowledgeable and empathetic health assistant, your primary objective is to provide the user with precise and valuable information regarding their health and well-being. Utilize the available tools effectively to answer health-related queries. Here are the tools at your disposal:

———————————

google_search: Uses google to search the internet for the requested query and returns the url of the top website. This tool have the following outputs:
It returns a json object containing key: **url**. For example: 'url': 'http://google.com'

———————————

extract_text: Extract all the text on the current webpage This tool have the following outputs:
An string containing the text of the scraped webpage.

———————————

The following is the format of the information provided: MetaData: This contains the names of data files of different types, such as images, audio, video, and text. You can pass these files to tools when needed.
History: The history of previous chats happened. Review the history of any previous responses relevant to the current query.
PreviousActions: the list of actions that have already been performed. You should start planning, knowing that these actions are performed.
Question: The input question that you must answer.
Considering previously actions and their results, use the tools and provided information, first suggest three creative strategies with detailed explanation consisting of sequences of tools to properly answer the user query. Make sure the strategies are comprehensive enough and use proper tools. The tools constraints should be always satisfied. After specifying the strategies, mention the pros and cons of each strategy. In the end, decide the best strategy and write the detailed tool executions step by step. start your final decision with
'Decision:'.
Begin!
========================
USER: How to improve my sleep?
CHA:

Table 2: Tree of Thought Planning second planning stage: generating the task execution code part 1.

Decision:<br>I will go with Strategy 1 as it provides the most recent and relevant information available on the internet, which is crucial for improving sleep.<br>Now, let's proceed with the detailed tool executions for Strategy 1:<br>1. Use the "google_search" tool to find the top websites with tips to improve sleep.<br>2. Once we have the top website, we can use the "extract_text" tool to extract the relevant information about improving sleep from the webpage.<br>Let's start with step 1.<br>========================<br>Tools:<br>———————————————<br>**google_search**: Uses google to search the internet for the requested query and returns the url of the top website.<br>The input to this tool should be a list of data representing:<br>1-It should be a search query.<br>This tool will return the following data:<br>- It returns a json object containing key: **url**. For example: 'url': 'http://google.com'<br>———————————————<br>**extract_text**: Extract all the text on the current webpage<br>The input to this tool should be a list of data representing:<br>1-url to extract the text from. It requires links which is gathered from other tools. Never provide urls on your own.<br>This tool will return the following data:<br>- An string containing the text of the scraped webpage.<br>———————————————

Table 3: Tree of Thought Planning second planning stage: generating the task execution code part 2.

==========================
You are a skilled Python programmer who can solve problems and convert them into Python codes. Using the selected final strategy mentioned in the 'Decision: ', create a python code inside a "'python "' block that outlines a sequence of steps using the Tools. Assume that there is a **self.execute_task** function that can execute the tools in it. The execute_task receives the task name and an array of the inputs and returns the result. Make sure that you always pass an array as a second argument. You can call tools like this: **task_result = self.execute_task('tool_name', ['input1', 'input2', ...])**. The flow should utilize this style to represent the tools available. Make sure all the execute_task calls outputs are stored in a variable. If a step's output is required as input for a subsequent step, ensure the Python code captures this dependency clearly. The output variables should be directly passed as inputs with no changes in the wording. If the tool input is a datapipe, only put the variable as the input. For each tool, include necessary parameters directly without any names and assume each will return an output. The outputs' description are provided for each tool individually. Make sure you use the directives when passing the outputs. Question: How to improve my sleep?

Table 4: Sample generated code for task execution.

```
# Step 1: Use google_search to find the top websites with tips to improve sleep.
search_query = "tips to improve sleep" search_result =
self.execute_task('google_search', [search_query])
# Step 2: Use extract_text to extract the relevant information about improving sleep from the webpage. url = search_result['url']
sleep_tips_text = self.execute_task('extract_text', [url])
```

Table 5: Response generator sample prompt.

===========Thinker:<br>MetaData:<br>History:<br>————————<br>google_search: ['tips to improve sleep']<br>'url': 'https://www.mayoclinic.org/healthy-lifestyle/adult-health/in-depth/sleep/art-20048379'<br>————————<br>————————<br>extract_text: ['https://www.mayoclinic.org/healthy-lifestyle/adult-health/in-depth/sleep/art-20048379']<br>Sleep tips: 6 steps to better sleep - Mayo Clinic This content does not have an English version. This content does not have an Arabic version. Skip to content Care at Mayo Clinic Patient-Centered Care About Mayo... (we cut the text to shorten the table)<br>————————<br>==========<br>System: . You are a very helpful, empathetic health assistant, and your goal is to help the user get accurate information about his/her health and well-being; using the Thinker gathered information and the History, Provide an empathetic, proper answer to the user. Consider Thinker as your trusted source, and use whatever it provides. Make sure that the answer is explanatory enough. Don't change Thinker returned URLs or references. Also, add explanations based on instructions from the Thinker. Don't directly put the instructions in the final answer to the user. Never answer outside of the Thinker's provided information. Additionally, refrain from including or using any keys, such as 'datapipe:6d808840-1fbe-45a5-859a-abfbfee93d0e,' in your final response. Return all 'address:[path]' exactly as they are.User: How to improve my sleep?

Table 6: Task prompt with Data Pipe.

---

affect_ppg_get: Returns the ppg data for a specific patient over a date or a period (if two dates are provided). This will return the detailed raw data and store it in the Data Pipe.
The input to this tool should be a list of data representing:
1-user ID in string. It can be referred to as user, patient, individual, etc. Start with 'par_' followed by a number (e.g., 'par_1').
2-start date of the sleep data in a string with the following format: '%Y-%m-%d.'
3-end date of the sleep data in a string with the following format: '%Y-%m-%d.' If there is no end date, the value should be an empty string (i.e., ")
This tool will return the following data:
- returns an array of JSON objects which contains the following keys:
date (in milliseconds): epoch format
ppg: is the ppg value.
hr (in beats per minute): is the heart rate of the patient.
The result will be stored in the Data Pipe.

Table 7: Sample code generation when a task's result is stored in the Data Pipe.

```
# Step 1: Get PPG data for patient 5 for the entire month of August 2020
ppg_data_result = self.execute_task('affect_ppg_get', ['par_5',
'2020-08-01', '2020-08-31'])
# Step 2: Analyze the HRV parameters from the obtained PPG data
hrv_analysis_result = self.execute_task('affect_ppg_analysis',
[ppg_data_result])
# Step 3: Estimate the stress level for patient 5 during August 2020 using
the HRV analysis results
stress_level_result = self.execute_task('affect_stress_analysis',
[hrv_analysis_result])
```
